# Comment on Fierro et al. Severe Hypotension, Bradycardia and Asystole after Sugammadex Administration in an Elderly Patient. *Medicina* 2021, *57*, 79

**DOI:** 10.3390/medicina58010122

**Published:** 2022-01-14

**Authors:** Orlando Sagliocco, Mauro Betelli

**Affiliations:** 1 Intensive Care Unit, Bolognini Hospital, ASST Bergamo Est, 24068 Seriate, Italy; 2 Internal Medicine Department, Bolognini Hospital, ASST Bergamo Est, 24068 Seriate, Italy; mauro.betelli@asst-bergamoest.it

We read with great interest the case report by Fierro et al. [1] about a cardiovascular arrest after administration of sugammadex—a gamma–cyclodextrin used in general anesthesia—in an elderly patient undergoing percutaneous nephrolithotripsy. The authors interpreted this event as a rare adverse drug reaction directly involving the cardiovascular function, but we suggest considering a particular kind of hypersensitivity reaction known as “Kounis syndrome”.

Fierro et al. stated themselves they could not definitively rule out anaphylaxis because clinical diagnostic criteria—according to the “Second symposium on the definition and management of anaphylaxis” [2]—could have been impaired during general anesthesia; in addition, neither tryptase levels nor allergy tests were performed. Nevertheless, they considered anaphylaxis less probable than a direct cardiovascular complication for the following reasons: (a) cases of marked bradycardia resulting in cardiac arrest are already reported in the literature and sugammadex data sheet; (b) the patient showed none of the common sugammadex-induced anaphylaxis signs (generalized skin rash, wheezing, bronchospasm, tachycardia); (c) the patient showed none of the common signs of generalized anaphylaxis (increased peak inspiratory pressures in mechanical ventilation, initial drop in EtCO2, facial or soft palate edema); (d) hemodynamics improved with atropine and ephedrine, while epinephrine was never administered.

However, relative bradycardia—defined as a severe form in which heart rate falls despite worsening hypotension—has been observed in a randomized controlled trial on venom immunotherapy and anaphylaxis by Brown et al. [3] They observed hypotensive reactions associated with relative bradycardia in a cohort of sixty-eight allergic volunteers exposed to sting challenge: eight developed hypotension with bradycardia, two of which in a severe form, one responding to atropine alone. The authors [4] postulated that while bradycardia may be a non-specific feature of severe hypovolaemic-distributive shock, relative bradycardia should be explained by the effect of anaphylactic mediators on the heart and/or nervous system and neurocardiogenic mechanisms. In recent decades, an increasing recognition of the human heart—in which mast-cell density is related to disease progression—as a major shock organ in some cases of anaphylaxis has been stated [4] and heart has been found to be both the source and a target of anaphylaxis mediators. Current evidence suggests [5] that eicosanoids and platelet activating factor (PAF) may influence the contractile function and contribute to myocardial depression and impaired ventricular function. Moreover, cases of severe reversible cardiac dysfunction associated with non-specific electrocardiogram changes and normal coronary arteries during anaphylaxis have been described since the 80s [6].

Kounis first described a syndrome characterized by the association of multiple acute coronary syndromes—including coronary spasm, acute myocardial infarction and stent thrombosis—with mast-cell and platelet activation in the setting of allergic/hypersensitivity reactions and anaphylactic/anaphylactoid insults. Cases are described in patients of any age or medical condition [7]; clinical presentation does not include typical anaphylactic signs such as skin rash, redness, erythematous wheal or urticaria [7,8]; a wide range of electrocardiographic abnormalities are reported [7,9]. Above all, cardiogenic shock and cardiac arrest developed, respectively, in 2.3% and 6.3% of the cases [9].

In a review by Tsur [8], fifteen cases of hypersensitivity reaction after sugammadex administration are reported; no previous exposure to this drug was reported in 80% (12/15) of the cases—leading to conjecture a sensitizing mechanism involving food cyclodextrins. Four cases (27%) did not meet anaphylaxis diagnostic criteria of “World Anaphylaxis Organization”, while nine cases (60%) showed hypotension. Even lab tests can be misleading: Bedirli et al. [10] described a case of sugammadex-induced severe anaphylaxis with biphasic hypotension and bronchospasm, despite serum Ig E and tryptase levels being within the normal range.

In 2018, the first report [11] of cardiac arrest following sugammadex-induced anaphylaxis was submitted: description of the case was similar to that of Fierro et al., but anaphylaxis could be confirmed through lab tests including serum tryptase levels. A 2020 sugammadex data sheet [12], mostly derived from post-marketing experience, reported cardiac disorders among other adverse drug reactions in the following way: (1) bradycardia and bradycardia with cardiac arrest; (2) rhythm abnormalities (marked atrial fibrillation, atrioventricular block, cardiac/cardiorespiratory arrest, electrocardiographic ST segment changes, supraventricular tachycardia/extrasystoles, tachycardia, ventricular fibrillation, ventricular tachycardia); (3) anaphylaxis associated with electrocardiographic ST segment changes, consistent with myocardial ischemia or coronary spasm.

Yanai and Ariyoshi [13] reported two cardiac arrests in the same patient, five months apart from one other and both related to sugammadex administration. The two episodes were different in several aspects (e.g., rhythm presentation: ventricular fibrillation vs. pulseless electric activity, electrocardiography pattern at return of spontaneous circulation: sinus tachycardia vs. diffuse ST segment depression, echocardiography: normal vs. severe left ventricular dysfunction). In the first case neither therapy nor specific tests were performed, while in the second one serum tryptase levels were elevated, the skin allergy test for sugammadex was positive and urgent coronary angiography revealed multiple coronary spasms (intracoronary nitroglycerin was administrated), thus prompting the diagnosis of type I Kounis syndrome. Nevertheless, the authors reasonably postulated that the first case was also a sugammadex-induced Kounis syndrome.

In conclusion, we suggest that in this case a diagnosis of Kounis or Kounis-like syndrome could also be strongly taken into account. This work highlights Kounis syndrome’s protean nature and the risk of underestimation, especially in the anesthesia setting, such as in the case described by Fierro et al. Moreover, diagnosis is challenging but essential, especially in case of acute coronary syndrome: even if treatment guidelines are still lacking [14], timely use of nitrates in type I subgroup can ensure better outcome than the usual outcome of common types of coronary acute syndrome [9]. Thus, conduction of large prospective trials to increase knowledge and awareness about this life-threatening disease is required.
